# SARS-CoV-2 Infection and New-Onset Type 2 Diabetes Among Pediatric Patients, 2020 to 2022

**DOI:** 10.1001/jamanetworkopen.2024.39444

**Published:** 2024-10-14

**Authors:** Margaret G. Miller, Pauline Terebuh, David C. Kaelber, Rong Xu, Pamela B. Davis

**Affiliations:** 1 Center for Artificial Intelligence in Drug Discovery, Case Western Reserve University School of Medicine, Cleveland, Ohio; 2 Center for Clinical Informatics Research and Education, The MetroHealth System and Departments of Medicine, Pediatrics, and Population and Quantitative Health Sciences, Case Western Reserve University, Cleveland, Ohio; 3 Center for Community Health Integration, Case Western Reserve University School of Medicine, Cleveland, Ohio

## Abstract

**Importance:**

In adults, diagnoses of new-onset type 2 diabetes (T2D) have increased following diagnosis with COVID-19, but whether this occurs in children is unclear.

**Objective:**

To determine whether risk of incident T2D diagnosis is increased during the 6 months after SARS-CoV-2 infection among children.

**Design, Setting, and Participants:**

This retrospective cohort study used electronic health records from the TriNetX analytics platforms between January 1, 2020, and December 31, 2022. Pediatric patients aged 10 to 19 years without preexisting diabetes were eligible for inclusion. Data were analyzed from August 15 to September 15, 2023, with supplemental analyses January 20 and August 8 to 13, 2024.

**Exposures:**

Diagnosis of COVID-19 or a non–COVID-19 respiratory infection.

**Main Outcomes and Measures:**

New diagnosis of T2D compared by risk ratios (RRs) and 95% CIs at 1, 3, and 6 months after index infection.

**Results:**

The main study population included 613 602 patients, consisting of 306 801 with COVID-19 (mean [SD] age at index, 14.9 [2.9] years; 52.8% female) and 306 801 with other respiratory infections (ORIs) but no documented COVID-19 (mean [SD] age at index, 14.9 [2.9] years; 52.6% female) after propensity score matching. Risk of a new diagnosis of T2D was significantly increased from day of infection to 1, 3, and 6 months after COVID-19 diagnosis compared with the matched cohort with ORIs (RR at 1 month, 1.55 [95% CI, 1.28-1.89]; RR at 3 months: 1.48 [95% CI, 1.24-1.76]; RR at 6 months: 1.58 [95% CI, 1.35-1.85]). Similar results were found in the subpopulation classified as having overweight or obesity (RR at 1 month: 2.07 [95% CI, 1.12-3.83]; RR at 3 months: 2.00 [95% CI, 1.15-3.47]; RR at 6 months: 2.27 [95% CI, 1.38-3.75]) and the hospitalized subpopulation (RR at 1 month: 3.10 [95% CI, 2.04-4.71]; RR at 3 months: 2.74 [95% CI, 1.90-3.96]; RR at 6 months: 2.62 [95% CI, 1.87-3.66]). Similar elevation in risk was found at 3 and 6 months when excluding patients diagnosed during the interval from the index date to 1 month after infection.

**Conclusions and Relevance:**

In this retrospective cohort study of children and adolescents aged 10 to 19 years, the risk of an incident diagnosis of T2D was greater following a COVID-19 diagnosis than in children diagnosed with ORIs. Further study is required to determine whether diabetes persists or reverses later in life.

## Key Points

**Question:**

Is a COVID-19 diagnosis associated with increased incident diagnoses of type 2 diabetes in children and adolescents?

**Findings:**

In this retrospective cohort study of 613 602 patients aged 10 to 19 years, there was an increased risk of new diagnoses of type 2 diabetes within 6 months following a diagnosis of COVID-19 compared with a diagnosis of other respiratory infections.

**Meaning:**

These findings suggest that understanding the role that SARS-CoV-2 plays in pediatric type 2 diabetes incidence will add an important component to consideration of the risks and benefits of preventing SARS-CoV-2 infection in children.

## Teaser

This cohort study assesses the risk of development of type 2 diabetes in pediatric patients aged 10 to 19 years after receiving a COVID-19 diagnosis compared with the risk after other non–COVID-19 respiratory infections during the first 2 years of the pandemic.

## Introduction

Infection with SARS-CoV-2 is associated with a wide range of subsequent chronic illness in patients, including diabetes.^1,2^ Among adults, a meta-analysis considering data from December 2019 through October 2022^3^ found an overall 66% higher risk of new-onset diabetes after SARS-CoV-2 infection; a second meta-analysis^4^ found that 12 of 14 studies found a significantly increased risk, ranging from 11% to 276%, for new-onset diabetes after SARS-CoV-2 infection. Several studies^4,5^ indicate that this risk is greater in male patients and in those with more severe SARS-CoV-2 infection.

While most studies included adults, the Centers for Disease Control and Prevention (CDC) reported an increased incidence of diabetes after COVID-19 among patients younger than 18 years based on health claims data but without distinguishing type 1 (T1D) from type 2 (T2D) diabetes.^6^ Subsequent studies,^7,8^ including a large study using electronic health records (EHRs), found an association between SARS-CoV-2 infection and risk of subsequent new-onset T1D in children. Another report^9^ documented increases in other disorders in children following COVID-19, especially autoimmune complications, such as post–COVID-19 conditions, multisystem inflammatory syndrome, or myocarditis. One study^10^ found that new cases of T2D, which may or may not have an autoimmune component, increased by 77.2% in the first year of the pandemic compared with the mean of the previous 2 years of the pandemic. However, the reported research on the incidence of T2D in children following SARS-CoV-2 infection is sparse, and most studies include only small study populations.^11^

Before the pandemic, rates of T2D in children were rising globally.^12^ Between 2001 to 2017, prevalence of T2D in children and adolescents aged 10 to 19 years (hereinafter referred to as children) increased from 0.34 to 0.67 per 1000 youths, a 95% relative increase.^13^ Though the rise in childhood obesity largely parallels this rise in T2D, insulin resistance in adolescents is multifactorial. These factors include inactivity, obesity, hormones related to growth and puberty, genetic factors, family history, sex, and race and ethnicity, among others.^12,14^ It is possible that infections could contribute to the development of T2D as well.

This study seeks to understand the risk of T2D development in pediatric patients aged 10 to 19 years after receiving a medically attended COVID-19 diagnosis compared with the risk after other non–COVID-19 respiratory infections (ORIs) during the first 2 years of the pandemic by using aggregated EHR data. Given the possibility of several factors contributing to effect modification, comparisons were also made for subpopulations of children based on body mass index (BMI; calculated as the weight in kilograms divided by the height in meters squared), disease severity, and sex.

## Methods

This retrospective cohort study used TriNetX, a global federated clinical data analytics platform for research.^15^ Specifically, the US collaborative network within TriNetX, which includes deidentified EHR data from over 100 million patients contributed by 60 large health care organizations across the country, was used. The network contains diverse representation of geographic areas, self-reported or clinician-observed race and ethnicity (included because these data are reported to be associated with COVID-19 diagnosis and severity^16^), age, income, and insurance types.^17^ Built-in analytic functions allow for analysis of patient-level data in aggregate form only, masking individual protected health information (see eMethods in Supplement 1 for details). The MetroHealth System Institutional Review Board has designated the use of deidentified aggregated data on the TriNetX platform in ways such as described in this report as not involving human participants; therefore, this study was exempt from review and the requirement for informed consent. The study design and results reporting followed the guidelines of the Strengthening the Reporting of Observational Studies in Epidemiology (STROBE) reporting guidelines.

The main study population consisted of pediatric patients aged 10 to 19 years at the time of the index event with an encounter diagnosis of a respiratory infection (COVID-19 or ORI) between January 1, 2020, and December 31, 2022. The age range was selected to correspond with that used by the CDC when reporting data on pediatric T2D from the SEARCH for Diabetes in Youth Study.^13,18^ Inclusion criteria for the COVID-19 cohort were similar to those of previous studies^19^: *International Statistical Classification of Diseases and Related Health Problems, Tenth Revision* (*ICD-10*), code for COVID-19 (U07.1) as an encounter diagnosis or a positive test result for SARS-CoV-2 as documented by the TriNetX curated code 9088 for the presence of SARS-CoV-2 and related RNA between January 1, 2020, and December 31, 2022 (see eMethods in Supplement 1 for codes included in the composite code), and no record of COVID-19 or positive SARS-CoV-2 RNA detection before the study period. Inclusion criteria in the ORI cohort required an encounter diagnosis of *ICD-10* codes J00 to J06 (other acute upper respiratory infections), J09 to J18 (influenza and pneumonia), or J20 to J22 (other acute lower respiratory infections), and no record of COVID-19 diagnosis, positive SARS-CoV-2 test result (between January 1, 2020 to December 31, 2022), or SARS-CoV-2 antibodies during the prevaccine period (prior to December 31, 2020) (see eMethods in Supplement 1 for details). Patients were excluded from both cohorts for *ICD-10* code E10 (T1D) or for having either *ICD-10* code E11 (T2D) or the TriNetX curated code 9037 for hemoglobin A_1c_ (HbA_1c_) or hemoglobin total level in blood of at least 6.5% at least 1 day before the indexing event (COVID-19 or ORI) (see eMethods in Supplement 1 for codes included in the composite code).

To assess potential effect modification by BMI, the subset of patients with overweight (pediatric BMI 85th to <95th percentile for age [Z68.53]) or obesity (≥95th percentile for age [Z68.54]) documented within 5 years prior to the index event were compared. Similarly, the subset of patients with COVID-19– or ORI-associated hospitalizations that were identified by an inpatient visit documented at any time from 1 week prior to 1 month after the index respiratory event were compared. Although the timing of hospitalization occurred in proximity to the respiratory infection, the contribution of that infection to the reason for hospitalization cannot be ascertained. An additional analysis stratified by sex was conducted. The details can be accessed in eMethods in Supplement 1.

Beyond assessing T2D as an outcome between COVID-19 or ORI diagnosis, this outcome was assessed between the cohort with COVID-19 and another nonviral cohort constructed to represent common noninfectious reasons for medical encounters in this population. *ICD-10* codes used for this cohort were L70.0 (acne vulgaris), S42 (fracture of shoulder and upper arm), S52 (fracture of forearm), Z01.0 (encounter for examination of eyes and vision), and Z46.0 (encounter for fitting and adjustment of spectacles and contact lenses).

T2D as an outcome was identified by the *ICD-10* encounter diagnosis E11. We measured cumulative risk of T2D following either COVID-19 or ORIs after time intervals of the same day to 1, 3, and 6 months. In acknowledging that T2D has metabolic disturbance occurring for some time prior to diagnosis, we conducted supplemental analyses excluding the acute period around the time of the respiratory illness (up to 1 month after) and excluding a 3-month period after the initial respiratory encounter diagnosis. The intervals of analysis constituted 1 to 6 months, 3 to 6 months, 1 to 9 months, and 3 to 9 months. Since diagnosis of T2D is often based on an elevated HbA_1c_ level, excluding patients diagnosed within 3 months of the index event was intended to help exclude patients whose HbA_1c_ level was elevated due to hyperglycemia that predated the respiratory illness. Similarly, supplemental analyses of patients with prediabetes were included. Details can be found in eMethods in Supplement 1.

### Statistical Analysis

Data were analyzed from August 15 to September 15, 2023, with supplemental analyses January 20 and August 8 to 13, 2024. Cohorts were propensity score matched (1:1 using nearest-neighbor greedy matching) for demographic characteristics, laboratory values for cholesterol level, risk factors for developing T2D, autoimmune risk factors, and various other *ICD-10* diagnoses that previous studies have found correlated with an increased risk of developing T2D (Table 1 includes the specific list).^20,21,22,23,24,25^ The matched autoimmune diseases were chosen from those that Hemminki et al^20^ found to be associated with an increased likelihood for T2D diagnoses in patients younger than 50 years. A standardized mean difference of less than 0.100 is considered a good match. Risk of new diagnoses of T2D after COVID-19 diagnosis was compared between the matched cohorts using risk ratios (RRs) and 95% CIs. Statistical analyses were conducted using the R statistical package, version 3.2.3 (R Program for Statistical Computing) that is embedded within the TriNetX analytics platform. Statistical significance was assessed between the matched cohorts using RRs and 2-sided 95% CIs, with significance defined as a 95% CI excluding 1.

**Table 1.** Baseline Characteristics of Main Study Population Before and After Propensity Score Matching^a^

Characteristic	Cohort before matching, No. (%)		SMD^b^	Cohort after matching, No. (%)		SMD^b^
	COVID-19 (n = 306 864)	ORI (n = 611 008)		COVID-19 (n = 306 801)	ORI (n = 611 008)	
Age when data accessed, mean (SD), y	16.9 (3.0)	16.4 (3.2)	0.14	16.9 (3.0)	16.8 (3.1)	0.006
Age at index, mean (SD), y^c^	14.9 (2.9)	14.3 (3.0)	0.20	14.9 (2.9)	14.9 (2.9)	0.004
Sex						
Female	161 972 (52.8)	327 166 (53.5)	0.02	161 940 (52.8)	161 483 (52.6)	0.003
Male	144 613 (47.1)	283 583 (46.4)	0.01	144 589 (47.1)	145 095 (47.3)	0.003
Unknown	279 (0.1)	259 (0.04)	0.02	272 (0.1)	223 (0.1)	0.03
Ethnicity^d^						
Hispanic or Latinx	46 340 (15.1)	81 512 (13.3)	0.05	46 326 (15.1)	47 454 (15.5)	0.01
Not Hispanic or Latinx	183 898 (59.9)	387 030 (63.3)	0.07	183 863 (59.9)	184 279 (60.1)	0.003
Unknown	76 626 (25.0)	142 466 (23.3)	0.04	76 612 (25.0)	75 068 (24.5)	0.010
Race^d^						
American Indian or Alaska Native	1293 (0.4)	2989 (0.5)	0.01	1292 (0.4)	1268 (0.4)	0.001
Asian	9327 (3.0)	18 097 (3.0)	0.04	9322 (3.0)	9591 (3.1)	0.005
Black or African American	58 056 (18.9)	85 815 (14.0)	0.13	58 023 (18.9)	53 890 (17.6)	0.03
Native Hawaiian or Other Pacific Islander	1503 (0.5)	2606 (0.4)	0.009	1503 (0.5)	1524 (0.5)	<0.001
White	174 155 (56.8)	382 781 (62.6)	0.12	174 141 (56.8)	175 285 (57.1)	0.008
Other^e^	62 530 (20.4)	118 720 (19.4)	0.02	62 520 (20.4)	65 243 (21.3)	0.02
Cholesterol level						
Measured	46 329 (15.1)	87 071 (14.3)	0.003	46 300 (15.1)	44 172 (14.4)	0.004
≥200 mg/dL	4974 (1.6)	8694 (1.4)	0.02	4956 (1.6)	4772 (1.6)	0.005
BMI						
Documented	54 535 (17.8)	142 921 (23.4)	0.14	54 519 (17.8)	54 923 (17.9)	0.003
≥95th percentile for age	22 901 (7.5)	48 704 (8.0)	0.02	22 890 (7.5)	23 385 (7.6)	0.006
85th to <95th percentile for age	12 891 (4.2)	33 998 (5.6)	0.06	12 885 (4.2)	12 970 (4.2)	0.001
Primary hypertension	4583 (1.5)	6646 (1.1)	0.04	4543 (1.5)	4254 (1.4)	0.008
Family history of diabetes	3526 (1.1)	6301 (1.0)	0.01	3517 (1.1)	3364 (1.1)	0.005
Pure hypercholesterolemia	2654 (0.9)	5364 (0.9)	0.001	2650 (0.9)	2430 (0.8)	0.008
Long-term use of systemic corticosteroids	1182 (0.4)	1529 (0.3)	0.03	1150 (0.4)	972 (0.3)	0.01
Other low-birth-weight newborn	1128 (0.4)	2526 (0.4)	0.007	1127 (0.4)	1037 (0.3)	0.005
Psoriasis	970 (0.3)	1872 (0.3)	0.002	968 (0.3)	877 (0.3)	0.005
Extremely low-birth-weight newborn	945 (0.3)	2057 (0.3)	0.005	944 (0.3)	885 (0.3)	0.003
Polycystic ovary syndrome	913 (0.3)	1500 (0.2)	0.01	912 (0.3)	918 (0.3)	<0.001
Autoimmune thyroiditis	884 (0.3)	1633 (0.3)	0.004	880 (0.3)	810 (0.3)	0.004
Celiac disease	780 (0.3)	1539 (0.3)	<0.001	780 (0.3)	705 (0.2)	0.005
Crohn disease	732 (0.2)	1085 (0.2)	0.01	724 (0.2)	671 (0.2)	0.004
Chronic rheumatic heart diseases	573 (0.2)	816 (0.1)	0.01	566 (0.2)	516 (0.2)	0.004
Secondary hypertension	481 (0.2)	505 (0.1)	0.02	448 (0.1)	376 (0.1)	0.006
Ulcerative colitis	450 (0.1)	599 (0.1)	0.01	440 (0.1)	404 (0.1)	0.003
Hyperthyroidism	397 (0.1)	663 (0.1)	0.006	396 (0.1)	349 (0.1)	0.004
Systemic lupus erythematosus	278 (0.1)	329 (0.1)	0.01	265 (0.1)	204 (0.1)	0.007
Immune thrombocytopenic purpura	258 (0.1)	379 (0.1)	0.008	251 (0.1)	228 (0.1)	0.003
Rheumatoid arthritis	115 (0.04)	180 (0.03)	0.004	113 (0.04)	83 (0.03)	0.005
Autoimmune hepatitis	84 (0.03)	74 (0.01)	0.01	77 (0.03)	59 (0.02)	0.004
Multiple sclerosis	77 (0.03)	84 (0.01)	0.008	76 (0.03)	62 (0.02)	0.003
Sjögren syndrome	61 (0.02)	88 (0.01)	0.004	60 (0.02)	55 (0.02)	0.001
Myasthenia gravis	41 (0.01)	46 (0.01)	0.006	41 (0.01)	30 (0.01)	0.003
Ankylosing spondylitis	37 (0.01)	49 (0.01)	0.004	35 (0.01)	28 (0.01)	0.002
Sarcoidosis	31 (0.01)	43 (0.01)	0.004	30 (0.01)	26 (0.01)	0.001
Systemic sclerosis	31 (0.01)	43 (0.1)	0.003	30 (0.01)	27 (0.01)	0.001
Infant of mother with gestational diabetes	24 (0.01)	40 (0.01)	0.002	24 (0.01)	12 (0.004)	0.005

Abbreviation: BMI, body mass index (calculated as the weight in kilograms divided by the height in meters squared); ORI, other respiratory infections; SMD, standardized mean difference.

SI conversion factors: To convert cholesterol to mmol/L, multiply by 0.0259.

^a^ Characteristics are measured up to 1 day prior to index infection.

^b^ An SMD greater than 0.1 is a threshold recommended for declaring imbalance.

^c^ Index event was diagnosis of COVID-19 or ORI.

^d^ Self-reported or clinician observed and included because they are reported to be associated with COVID-19 diagnosis and severity.

^e^ Includes patients identifing as multiracial or a race that is not listed in the aforementioned options.

## Results

**Figure 1.**
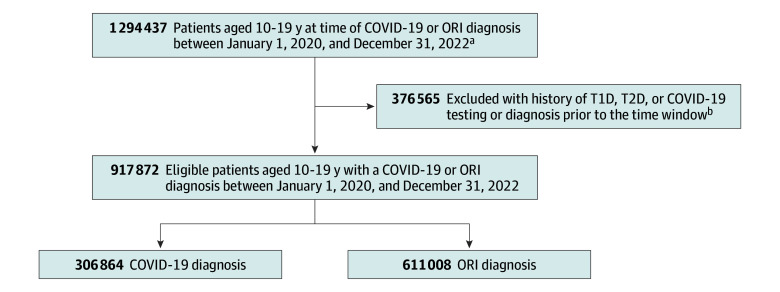
Flow Diagram of Cohort Construction for the Main Study Population ORI indicates other respiratory infection; T1D, type 1 diabetes; and T2D, type 2 diabetes. ^a^This number was obtained from an analysis conducted in August 2024. ^b^For the COVID-19 cohort, prior to the time window refers to prior to December 31, 2019. For the ORI cohort, prior to the time window refers to prior to June 30, 2023.

The overall study population included pediatric patients aged 10 to 19 years without prior documentation of preexisting diabetes or elevated HbA_1c_ levels and with EHR-documented COVID-19 (n = 306 864) or an ORI (n = 611 008) between January 1, 2020, and December 31, 2022 (Figure 1). After propensity score matching on baseline characteristics (Table 1), 613 602 patients were included overall, with 306 801 patients in each cohort. The COVID-19 cohort included 52.8% female and 47.1% male patients (0.1% unknown sex), with a mean (SD) index age of 14.9 (2.9) years. In terms of race and ethnicity, 0.4% were American Indian or Alaska Native; 3.0% Asian; 18.9% Black; 15.1% Hispanic; 0.5% Native Hawaiian or Other Pacific Islander; 56.8% White; and 20.4% other race (including multiracial or a category not listed). The ORI cohort included 52.6% female and 47.3% male patients (0.1% unknown sex), with a mean (SD) index age of 14.9 (2.9) years. In terms of race and ethnicity, 0.4% were American Indian or Alaska Native; 3.1% Asian; 17.6% Black; 15.5% Hispanic; 0.5% Native Hawaiian or Other Pacific Islander; 57.1% White; and 21.3% other.

Table 2 summarizes patient counts as well as the cumulative relative risk of developing T2D after COVID-19 vs ORI for periods from the day of infection to 1, 3, and 6 months. At each point, patients with COVID-19 had a significantly elevated risk of developing T2D compared with patients with ORI (RR at 1 month: 1.55 [95% CI, 1.28-1.89]; RR at 3 months: 1.48 [95% CI, 1.24-1.76]; RR at 6 months: 1.58 [95% CI, 1.35-1.85]).

**Table 2.** Relative Risk of New Diagnosis of T2D Among Patients Aged 10-19 Years From the Same Day to 1, 3, and 6 Months After COVID-19 vs ORI Diagnosis

Time since index infection	Patients with new T2D, No. (%)^a^		RR (95% CI)
	Cohort with COVID-19	Cohort with ORI	
**All patients**			
No. of patients	306 801	306 801	NA
1 mo	253 (0.08)	163 (0.05)	1.55 (1.28-1.89)
3 mo	310 (0.10)	210 (0.07)	1.48 (1.24-1.76)
6 mo	398 (0.13)	252 (0.08)	1.58 (1.35-1.85)
**Patients with overweight or obesity** ^b^			
No. of patients	16 496	16 496	NA
1 mo	31 (0.19)	15 (0.09)	2.07 (1.12-3.83)
3 mo	38 (0.23)	19 (0.12)	2.00 (1.15-3.47)
6 mo	50 (0.30)	22 (0.13)	2.27 (1.38-3.75)
**Hospitalized patients** ^c^			
No. of patients	13 653	13 653	NA
1 mo	90 (0.66)	29 (0.21)	3.10 (2.04-4.71)
3 mo	107 (0.78)	39 (0.29)	2.74 (1.90-3.96)
6 mo	123 (0.90)	47 (0.34)	2.62 (1.87-3.66)

Abbreviations: NA, not applicable; ORI, other respiratory infection; RR, relative risk; T2D, type 2 diabetes.

^a^ Indicates new documentation of T2D by *International Statistical Classification of Diseases and Related Health Problems, Tenth Revision*, code.

^b^ Overweight or obesity inclusion based on at least 1, and the most recent, pediatric body mass index measurements of the 85th percentile or greater for age documented during prior 5 years.

^c^ Based on documentation of inpatient visit during the 1 week before to 1 month after diagnosis of index infection.

The subpopulation of pediatric patients aged 10 to 19 years with overweight or obesity, without prior documentation of preexisting diabetes or elevated HbA_1c_ levels, and with an EHR-documented COVID-19 infection (n = 16 5382) or an ORI (n = 43 151) between January 1, 2020, and December 31, 2022, included 16 469 patients in each cohort after propensity matching for baseline characteristics (eTable 1 in Supplement 1). The subpopulation of inpatient pediatric patients aged 10 to 19 years without prior documentation of preexisting diabetes or elevated HbA_1c_ levels and with an EHR-documented COVID-19 infection (n = 14 094) or ORI (n = 22 559) between January 1, 2020, and December 31, 2022, included 14 014 pediatric patients in each cohort after propensity matching for baseline characteristics (eTable 2 in Supplement 1). See eMethods in Supplement 1 for information about the baseline characteristics used for propensity matching for these subpopulation cohorts.

Among patients with 1 or more encounter diagnoses for a BMI of overweight or obesity, COVID-19 in comparison with ORI was associated with a significantly increased risk of developing T2D at each follow-up point (RR at 1 month: 2.07 [95% CI, 1.12-3.83]; RR at 3 months: 2.00 [95% CI, 1.15-3.47]; RR at 6 months: 2.27 [95% CI, 1.38-3.75]) (Table 2). When comparison was restricted to a subpopulation of pediatric patients who had an inpatient encounter within 1 month of documented COVID-19 infection or ORI, a similarly increased risk of developing T2D was seen at all points (RR at 1 month: 3.10 [95% CI, 2.04-4.71]; RR at 3 months: 2.74 [95% CI, 1.90-3.96]; RR at 6 months: 2.62 [95% CI, 1.87-3.66]) (Table 2).

**Figure 2.**
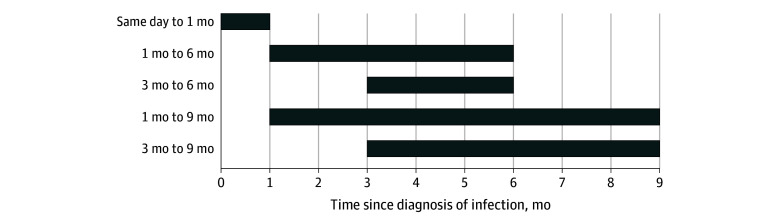
Time Intervals for Outcome Analysis Includes patients aged 10 to 19 years at various time intervals after COVID-19 vs other respiratory infection (ORI) diagnosis. Patients receiving an encounter diagnosis of T2D soon after the index infection of COVID-19 or ORI may have had preexisting diabetes that was newly discovered. Supplemental analyses were conducted to exclude patients with an encounter diagnosis of T2D within 1 to 3 months after the index event.

Table 3 shows the results when excluding patients whose initial diagnosis of T2D was made in the acute period (up to 1 month after) or up to 3 months after the respiratory infection encounter diagnosis (a visual depiction can be seen in Figure 2). A significantly increased risk of T2D after COVID-19 diagnosis compared with a non–COVID-19 respiratory infection was found at all time frames (RR range, 1.34 [95% CI, 1.08-1.66] at 1 to 9 months to 1.63 [95% CI, 1.14-2.33] at 3 to 6 months). Female and male patients did not differ in their risk of subsequent diagnosis of T2D following COVID-19 diagnosis (eTable 3 in Supplement 1). In analyses comparing the COVID-19 and ORI cohorts with prediabetes, as well as analyses comparing the COVID-19 cohort to the cohort with nonviral illness, estimates were similar to those for the main study population (eTables 5 and 7 in Supplement 1).

**Table 3.** Relative Risk of New Diagnosis of T2D Among Patients Aged 10-19 Years at Various Time Intervals After COVID-19 vs ORI Diagnosis

Time since index infection	Patients with T2D, No. (%)		RR (95% CI)
	Cohort with COVID-19 (n = 326 364)	Cohort with ORI (n = 326 364)	
Same day to 1 mo	267 (0.08)	184 (0.06)	1.45 (1.20-1.75)
1 mo to 6 mo	129 (0.04)^a^	88 (0.03)^b^	1.47 (1.12-1.92)
3 mo to 6 mo	78 (0.02)^c^	48 (0.01)^d^	1.63 (1.14-2.33)
1 mo to 9 mo	195 (0.06)^a^	146 (0.04)^b^	1.34 (1.08-1.66)
3 mo to 9 mo	144 (0.04)^c^	106 (0.03)^d^	1.36 (1.06-1.75)

Abbreviations: ORI, other respiratory infection; RR, relative risk; T2D, type 2 diabetes.

^a^ Excludes 360 patients with the outcome prior to the time window (n = 326 004).

^b^ Excludes 190 patients with the outcome prior to the time window (n = 326 174).

^c^ Excludes 411 patients with the outcome prior to the time window (n = 325 953).

^d^ Excludes 230 patients with the outcome prior to the time window (n = 326 134).

## Discussion

In this cohort study, pediatric patients aged 10 to 19 years with a COVID-19 diagnosis were significantly more likely to be diagnosed with T2D during the subsequent 6 months than propensity score–matched children who had ORIs during the first 3 years of the COVID-19 pandemic. This increased RR was present whether the analysis began on the date of diagnosis or 1 or 3 months after the diagnosis, which eliminated the risk of reverse causality. This observation is consistent with prior data in the literature for adults, which indicates an increased risk for a T2D diagnosis following a SARS-CoV-2 infection, and with the results of smaller studies in Germany and in the US.^26,27,28^ The increased RR for T2D diagnosis following COVID-19 diagnosis was also seen in the subset of patients who were hospitalized, as well as in those who had BMI classified as overweight or obese. Some of the studies conducted with adults reported increased risk for male patients,^5^ but this was not observed in our study.

To test whether the new diagnoses of T2D are increased in part because of bias of ascertainment, since symptomatic COVID-19 may bring to medical attention children who already have undiagnosed T2D, we performed additional analyses excluding T2D diagnoses made within 1 and 3 months of the respiratory illness. Cumulative risk for T2D after COVID-19 was elevated in comparison to ORI even when considering diagnoses initially documented during periods beginning 1 or 3 months after the onset of the respiratory event. Thus, although some of the T2D diagnoses could have been preexisting and simply were discovered during the encounter for infection, a substantial portion of the cases follow the infection. Diagnoses made in the first month following infection may well represent preexisting disease, since the diagnosis of T2D is often confirmed by HbA_1c_ measurements, which use mean blood glucose levels over the life span of the erythrocyte, or over 3 to 4 months. It will be important to test whether patients diagnosed soon after COVID-19 infection continue to meet diagnostic criteria for T2D, to separate patients who became hyperglycemic only during the metabolic stress of infection.

Our retrospective observational study cannot identify causation, but several possible causes of T2D might be affected by SARS-CoV-2. The additional metabolic stress imposed by COVID-19 may have pushed frank disease in an already susceptible child. In addition, attention has focused recently on possible autoimmune components of T2D, and it has been reported that genetically susceptible children have increased development of anti–β cell antibodies following COVID-19.^29,30^ In addition, SARS-CoV-2 may have the ability to selectively infect human pancreatic β cells,^31^ and if this triggers apoptosis, the ability of the pancreas to secrete insulin may be impaired. Though T2D is usually considered to be a disease of insulin resistance rather than insulin lack, for newly diagnosed patients, the origin may not be entirely clear or confined to a single pathobiologic cause.

Although the excess risk for a new diagnosis of T2D following COVID-19 infection compared with children who had ORIs, including in those who were hospitalized or classified as having overweight or obesity, is modest (4.8 per 10 000 in the general population but 17.0 per 10 000 among children with overweight or obesity), it represents a substantial population and cost burden.^32^ The estimated cost of diabetes in the US in 2022 was $412.9 billion, with $306.6 billion in direct medical costs and $106.3 billion in indirect costs due to disability, absenteeism, and lost productivity.^32^ Individuals with diabetes face annual mean medical expenses of $19 736 per year, about 2.6 times higher than those who do not have diabetes. For pediatric patients, the population we studied, the length of time that they will experience increased costs and morbidity is even greater than for adults. In addition, children may have a more severe T2D disease process than adults, and complications of T2D come even sooner in those who are diagnosed as children.^12,33,34^ Moreover, new drugs and interventions to control T2D such as weight control are more available.

### Limitations

This study has some limitations. Some potential contributing factors are difficult to assess, since some important variables are not consistently recorded in the EHR, such as socioeconomic status, BMI, insulin resistance, or vaccinations, which often occur outside health care organizations. We did not match our patients with obesity and overweight for obesity-specific treatments, which might have influenced the outcome; however, few children received drugs during this period. Race and ethnicity are self-reported or recorded by clinician observation and are missing in some patient EHR documentation. In addition, CDC data indicate that by late 2022, when our data collection ended, over 96% of children were serologically positive for natural infection, and there were about 50% of children positive by December 2021.^35^ Therefore, for patients with more recent medically attended COVID-19, the matched control group was more likely to include patients with undocumented or non–medically attended SARS-CoV-2 infections. This contamination could reduce our likelihood of detecting a true impact of SARS-CoV-2 infection on subsequent T2D diagnosis, so any association we detect may well be underestimated. EHRs are subject to overdiagnosis, underdiagnosis, or misdiagnosis, especially related to *ICD-10* codes, and this limitation is important to consider in relation to the results obtained for this study. There are some potential confounding factors that are not measurable or reported in this deidentified EHR dataset, such as the trajectory of weight gain in children with obesity, their activity level, or emotional stress. Finally, a retrospective observational study can identify associations but not determine causation. It can, however, prompt prospective studies that can be definitive.

## Conclusions

In this cohort study, pediatric patients aged 10 to 19 years had increased risk for a new diagnosis of T2D following COVID-19 infection compared with children who had ORIs, and this was true for those who were hospitalized and for those classified as having overweight or obesity. Understanding the role that SARS-CoV-2 plays in pediatric T2D incidence will add an important component to consideration of the risks and benefits of preventing SARS-CoV-2 infection in children.

## Notes

Supplement 1. **eMethods.** Study Population and Inclusion and Exclusion Codes **eTable 1.** Baseline Characteristics of Pediatric Patient Cohorts With Overweight or Obesity Before and After Propensity Score Matching **eTable 2.** Characteristics of Inpatient Pediatric Patient Cohorts Before and After Propensity Score Matching **eTable 3.** Comparison of Risk of New Diagnoses of T2D in Patients Aged 10 to 19 Years by Sex (Male vs Female) at 1, 3, and 6 Months From the Same Day of COVID-19 Diagnosis **eTable 4.** Characteristics of Pediatric Patient Cohorts With Prediabetes Before and After Propensity Score Matching **eTable 5.** Comparison of Risk of New Diagnoses of T2D in Patients Aged 10 to 19 Years With Prediabetes at 1, 3, and 6 Months From the Same Day of Respiratory Illness Diagnosis **eTable 6.** Baseline Characteristics of Pediatric Patient Cohorts With Viral and Nonviral Diagnoses Before and After Propensity Score Matching **eTable 7.** Comparison of Risk of New Diagnoses of T2D in Patients Aged 10 to 19 Years at 1, 3, and 6 Months From the Same Day COVID-19 Diagnosis or Nonviral Visit

Supplement 2.
Data Sharing Statement

## References

[1] Alyammahi SK, Abdin SM, Alhamad DW, Elgendy SM, Altell AT, Omar HA. The dynamic association between COVID-19 and chronic disorders: an updated insight into prevalence, mechanisms and therapeutic modalities. Infect Genet Evol. 2021;87:104647. doi:10.1016/j.meegid.2020.104647 33264669 PMC7700729

[2] Lopez-Leon S, Wegman-Ostrosky T, Perelman C, . More than 50 long-term effects of COVID-19: a systematic review and meta-analysis. Res Sq. 2021; 10.1038/s41598-021-95565-8 PMC8352980 34373540

[3] Ssentongo P, Zhang Y, Witmer L, Chinchilli VM, Ba DM. Association of COVID-19 with diabetes: a systematic review and meta-analysis. Sci Rep. 2022;12(1):20191. doi:10.1038/s41598-022-24185-7 36418912 PMC9684130

[4] Harding JL, Oviedo SA, Ali MK, . The bidirectional association between diabetes and long–COVID-19—a systematic review. Diabetes Res Clin Pract. 2023;195:110202. doi:10.1016/j.diabres.2022.110202 36496030 PMC9727969

[5] Naveed Z, Velásquez García HA, Wong S, . Association of COVID-19 infection with incident diabetes. JAMA Netw Open. 2023;6(4):e238866. doi:10.1001/jamanetworkopen.2023.8866 37071420 PMC10114057

[6] Barrett CE, Koyama AK, Alvarez P, . Risk for newly diagnosed diabetes >30 days after SARS-CoV-2 infection among persons aged <18 years—United States, March 1, 2020-June 28, 2021. MMWR Morb Mortal Wkly Rep. 2022;71(2):59-65. doi:10.15585/mmwr.mm7102e2 35025851 PMC8757617

[7] Kendall EK, Olaker VR, Kaelber DC, Xu R, Davis PB. Association of SARS-CoV-2 infection with new-onset type 1 diabetes among pediatric patients from 2020 to 2021. JAMA Netw Open. 2022;5(9):e2233014. doi:10.1001/jamanetworkopen.2022.33014 36149658 PMC9508649

[8] Marks BE, Khilnani A, Meyers A, . Increase in the diagnosis and severity of presentation of pediatric type 1 and type 2 diabetes during the COVID-19 pandemic. Horm Res Paediatr. 2021;94(7-8):275-284. doi:10.1159/000519797 34564073 PMC8805060

[9] Rao S, Gross RS, Mohandas S, . Postacute sequelae of SARS-CoV-2 in children. Pediatrics. 2024;153(3):e2023062570. doi:10.1542/peds.2023-062570 38321938 PMC10904902

[10] Magge SN, Wolf RM, Pyle L, ; COVID-19 and Type 2 Diabetes Consortium. The coronavirus disease 2019 pandemic is associated with a substantial rise in frequency and severity of presentation of youth-onset type 2 diabetes. J Pediatr. 2022;251:51-59.e2. doi:10.1016/j.jpeds.2022.08.010 35985535 PMC9383958

[11] Mefford MT, Wei R, Lustigova E, Martin JP, Reynolds K. Incidence of diabetes among youth before and during the COVID-19 pandemic. JAMA Netw Open. 2023;6(9):e2334953. doi:10.1001/jamanetworkopen.2023.34953 37733344 PMC10514735

[12] Shah AS, Nadeau KJ, Dabelea D, Redondo MJ. Spectrum of phenotypes and causes of type 2 diabetes in children. Annu Rev Med. 2022;73:501-515. doi:10.1146/annurev-med-042120-012033 35084995 PMC9022328

[13] Lawrence JM, Divers J, Isom S, ; SEARCH for Diabetes in Youth Study Group. Trends in prevalence of type 1 and type 2 diabetes in children and adolescents in the US, 2001-2017. JAMA. 2021;326(8):717-727. doi:10.1001/jama.2021.11165 34427600 PMC8385600

[14] Weiss R, Taksali SE, Tamborlane WV, Burgert TS, Savoye M, Caprio S. Predictors of changes in glucose tolerance status in obese youth. Diabetes Care. 2005;28(4):902-909. doi:10.2337/diacare.28.4.902 15793193

[15] Palchuk MB, London JW, Perez-Rey D, . A global federated real-world data and analytics platform for research. JAMIA Open. 2023;6(2):ooad035. doi:10.1093/jamiaopen/ooad035 37193038 PMC10182857

[16] Centers for Disease Control and Prevention. Health disparities: provisional death counts for COVID-19, race and Hispanic origin (2023). Accessed September 7, 2024. https://www.cdc.gov/nchs/nvss/vsrr/covid19/health_disparities.htm

[17] TriNetX. What is the geographic spread of HCOs in TriNetX downloadable datasets? Accessed September 7, 2024. https://trinetx.com/real-world-data/

[18] Divers J, Mayer-Davis EJ, Lawrence JM, . Trends in incidence of type 1 and type 2 diabetes among youths—selected counties and Indian reservations, United States, 2002-2015. MMWR Morb Mortal Wkly Rep. 2020;69(6):161-165. doi:10.15585/mmwr.mm6906a3 32053581 PMC7017961

[19] Taquet M, Geddes JR, Husain M, Luciano S, Harrison PJ. 6-month neurological and psychiatric outcomes in 236 379 survivors of COVID-19: a retrospective cohort study using electronic health records. Lancet Psychiatry. 2021;8(5):416-427. doi:10.1016/S2215-0366(21)00084-5 33836148 PMC8023694

[20] Hemminki K, Liu X, Försti A, Sundquist J, Sundquist K, Ji J. Subsequent type 2 diabetes in patients with autoimmune disease. Sci Rep. 2015;5(1):13871. doi:10.1038/srep13871 26350756 PMC4563366

[21] Kyrou I, Tsigos C, Mavrogianni C, ; Feel4Diabetes-study Group. Sociodemographic and lifestyle-related risk factors for identifying vulnerable groups for type 2 diabetes: a narrative review with emphasis on data from Europe. BMC Endocr Disord. 2020;20(suppl 1):134. doi:10.1186/s12902-019-0463-3 32164656 PMC7066728

[22] Gambineri A, Patton L, Altieri P, . Polycystic ovary syndrome is a risk factor for type 2 diabetes: results from a long-term prospective study. Diabetes. 2012;61(9):2369-2374. doi:10.2337/db11-1360 22698921 PMC3425413

[23] Knop MR, Geng TT, Gorny AW, . Birth weight and risk of type 2 diabetes mellitus, cardiovascular disease, and hypertension in adults: a meta-analysis of 7 646 267 participants from 135 studies. J Am Heart Assoc. 2018;7(23):e008870. doi:10.1161/JAHA.118.008870 30486715 PMC6405546

[24] Hussein WN, Mohammed ZM, Mohammed AN. Identifying risk factors associated with type 2 diabetes based on data analysis. Measur Sens. 2022;24:100543. doi:10.1016/j.measen.2022.100543

[25] Holder T, Giannini C, Santoro N, . A low disposition index in adolescent offspring of mothers with gestational diabetes: a risk marker for the development of impaired glucose tolerance in youth. Diabetologia. 2014;57(11):2413-2420. doi:10.1007/s00125-014-3345-2 25168408

[26] Denzer C, Rosenbauer J, Klose D, ; DPV Initiative. Is COVID-19 to blame? trends of incidence and sex ratio in youth-onset type 2 diabetes in Germany. Diabetes Care. 2023;46(7):1379-1387. doi:10.2337/dc22-2257 37140887

[27] Sasidharan Pillai S, Has P, Quintos JB, . Incidence, severity, and presentation of type 2 diabetes in youth during the first and second year of the COVID-19 pandemic. Diabetes Care. 2023;46(5):953-958. doi:10.2337/dc22-1702 36637859

[28] Schmitt JA, Ashraf AP, Becker DJ, Sen B. Changes in type 2 diabetes trends in children and adolescents during the COVID-19 pandemic. J Clin Endocrinol Metab. 2022;107(7):e2777-e2782. doi:10.1210/clinem/dgac209 35377436 PMC8992346

[29] Koufakis T, Dimitriadis G, Metallidis S, Zebekakis P, Kotsa K. The role of autoimmunity in the pathophysiology of type 2 diabetes: looking at the other side of the moon. Obes Rev. 2021;22(8):e13231. doi:10.1111/obr.13231 33682984

[30] de Candia P, Prattichizzo F, Garavelli S, . Type 2 diabetes: how much of an autoimmune disease? Front Endocrinol (Lausanne). 2019;10:451. doi:10.3389/fendo.2019.00451 31333589 PMC6620611

[31] Wu CT, Lidsky PV, Xiao Y, . SARS-CoV-2 infects human pancreatic β cells and elicits β cell impairment. Cell Metab. 2021;33(8):1565-1576.e5. doi:10.1016/j.cmet.2021.05.013 34081912 PMC8130512

[32] Parker ED, Lin J, Mahoney T, . Economic costs of diabetes in the U.S. in 2022. Diabetes Care. 2024;47(1):26-43. doi:10.2337/dci23-0085 37909353

[33] Arslanian S, Kim JY, Nasr A, . Insulin sensitivity across the lifespan from obese adolescents to obese adults with impaired glucose tolerance: who is worse off? Pediatr Diabetes. 2018;19(2):205-211. doi:10.1111/pedi.12562 28726334

[34] Hamman RF, Bell RA, Dabelea D, ; SEARCH for Diabetes in Youth Study Group. The SEARCH for Diabetes in Youth study: rationale, findings, and future directions. Diabetes Care. 2014;37(12):3336-3344. doi:10.2337/dc14-0574 25414389 PMC4237981

[35] Centers for Disease Control and Prevention. COVID data tracker. Updated daily. September 15, 2023. https://covid.cdc.gov/covid-data-tracker/#pediatric-seroprevalence

