# Supplementary material for: SARS-CoV-2 Infection and New-Onset Type 2 Diabetes Among Pediatric Patients, 2020 to 2022
Source: JAMA Netw Open. 2024 Oct 14;7(10):e2439444. doi: 10.1001/jamanetworkopen.2024.39444 (PMC11581647; doi:10.1001/jamanetworkopen.2024.39444)
Supplement: Supplement 1. — eMethods. Study Population and Inclusion and Exclusion Codes eTable 1. Baseline Characteristics of Pediatric Patient Cohorts With Overweight or Obesity Before and After Propensity Score Matching eTable 2. Characteristics of Inpatient Pediatric Patient Cohorts Before and After Propensity Score Matching eTable 3. Comparison of Risk of New Diagnoses of T2D in Patients Aged 10 to 19 Years by Sex (Male vs Female) at 1, 3, and 6 Months From the Same Day of COVID-19 Diagnosis eTable 4. Characteristics of Pediatric Patient Cohorts With Prediabetes Before and After Propensity Score Matching eTable 5. Comparison of Risk of New Diagnoses of T2D in Patients Aged 10 to 19 Years With Prediabetes at 1, 3, and 6 Months From the Same Day of Respiratory Illness Diagnosis eTable 6. Baseline Characteristics of Pediatric Patient Cohorts With Viral and Nonviral Diagnoses Before and After Propensity Score Matching eTable 7. Comparison of Risk of New Diagnoses of T2D in Patients Aged 10 to 19 Years at 1, 3, and 6 Months From the Same Day COVID-19 Diagnosis or Nonviral Visit [file jamanetwopen-e2439444-s001.pdf]

Supplementary Online Content

Miller MG, Terebuh P, Kaelber DC, Xu R, Davis PB. SARS-CoV-2 infection and new-onset type 2 diabetes among pediatric patients, 2020 to 2022. *JAMA Netw Open*. 2024;7(10):e2439444. doi:10.1001/jamanetworkopen.2024.39444

eMethods. Study Population and Inclusion and Exclusion Codes

eTable 1. Baseline Characteristics of Pediatric Patient Cohorts With Overweight or Obesity Before and After Propensity Score Matching

eTable 2. Characteristics of Inpatient Pediatric Patient Cohorts Before and After Propensity Score Matching

eTable 3. Comparison of Risk of New Diagnoses of T2D in Patients Aged 10 to 19 Years by Sex (Male vs Female) at 1, 3, and 6 Months From the Same Day of COVID-19 Diagnosis

eTable 4. Characteristics of Pediatric Patient Cohorts With Prediabetes Before and After Propensity Score Matching

eTable 5. Comparison of Risk of New Diagnoses of T2D in Patients Aged 10 to 19 Years With Prediabetes at 1, 3, and 6 Months From the Same Day of Respiratory Illness Diagnosis

eTable 6. Baseline Characteristics of Pediatric Patient Cohorts With Viral and Nonviral Diagnoses Before and After Propensity Score Matching

eTable 7. Comparison of Risk of New Diagnoses of T2D in Patients Aged 10 to 19 Years at 1, 3, and 6 Months From the Same Day COVID-19 Diagnosis or Nonviral Visit

This supplementary material has been provided by the authors to give readers additional information about their work.

© 2024 Miller MG et al. *JAMA Network Open*.

eMethods. Study Population and Inclusion and Exclusion Codes

The data used in this study were accessed from August to September 2023 with supplemental analyses in January 2024 and August 2024 from the TriNetX US Collaborative Network without the use of natural language processing. TriNetX data is continually and regularly refreshed, leading to changes in composition of cohorts when they are collected at different times. TriNetX is a global federated real-world data and analytics platform for research that provides access to electronic health records (diagnoses, procedures, medications, laboratory values, genomic information) from over 100 million patients from 60 healthcare organizations. This retrospective cohort study is exempt from informed consent. The data reviewed is a secondary analysis of existing data, does not involve intervention or interaction with human subjects, and is de-identified per the de-identification standard defined in Section §164.514(a) of the HIPAA Privacy Rule. The process by which the data is de-identified is attested to through a formal determination by a qualified expert as defined in Section §164.514(b)(1) of the HIPAA Privacy Rule. This formal determination by a qualified expert refreshed on December 2020. TriNetX is certified to the ISO 27001:2013 standard and maintains an Information Security Management System (ISMS) to ensure the protection of the healthcare data it has access to and to meet the requirements of the HIPAA Security Rule. Some HCOs that contribute to the network date-shift individual EHRs from 1 to 365 days on the calendar as an additional privacy protection, however the relative timing within each health record is maintained. The MetroHealth System in Cleveland, Ohio, IRB has determined that research using TriNetX in ways such as described in this manuscript is not Human Subject Research and therefore exempt from IRB review.

The TriNetX platform de-identifies and aggregates electronic health record (EHR) data from 60 contributing healthcare systems, most of which are large academic medical institutions with both inpatient and outpatient facilities at multiple locations, across all 50 states in the US. Patient EHR data includes information from hospitals, primary care, and specialty treatment providers, covering diverse geographic locations, age groups, racial and ethnic groups, income levels and insurance types including various commercial insurances, governmental insurance (Medicare and Medicaid), self-pay/uninsured, worker compensation insurance, and military/VA insurance among others. Race and ethnicity data in TriNetX is derived from self-reports or provider observaitons in the clinical EHR systems, which is then mapped to the following categories according to census categories: (1) Race: Asian, American Indian or Alaskan Native, Black or African American, Native Hawaiian or Other Pacific Islander, White, Unknown race; and (2) Ethnicity: Hispanic or Latino, Not Hispanic or Latino, Unknown Ethnicity.

COVID-19 Cohort: Inclusion and Exclusion Criteria Codes

Included if either of the codes below from January 1, 2020 to December 31, 2022:

U07.1: COVID-19 – 10-19 years
9088 (TNX curated): SARS coronavirus 2 and related RNA [Presence] – 10-19 years and positive

Excluded if any of the code below at least 1 day before the first instance of the code above:

E11: Type 2 diabetes mellitus
9037 (TNX curated): Hemoglobin A1c/Hemoglobin total in blood ≥ 6.5%

Excluded if any code below occurred anytime:

E10: Type 1 diabetes

Excluded if any of the code below on or before December 31, 2019 in order to exclude patients who may have had COVID-19 and their EHRs were date-shifted to before the onset of the pandemic:

B97.29: Other coronavirus as the cause of diseases classified elsewhere
J12.82: Pneumonia due to COVID-19
J12.81: Pneumonia due to SARS-associated coronavirus

B34.2: Coronavirus infection, unspecified
U07.1: COVID-19
U07.2: COVID-19, virus not identified (WHO)
9088 (TNX curated): SARS coronavirus 2 and related RNA [Presence] – positive, ever
LOINC 94558-4: SARS-CoV-2 (COVID-19) Ag [Presence] in Respiratory specimen by Rapid immunoassay – positive, ever
LOINC 97097-0: SARS-CoV-2 (COVID-19) Ag [Presence] in Upper respiratory specimen by Rapid immunoassay – positive, ever
LOINC 95209-3: SARS coronavirus + SARS coronavirus 2 Ag [Presence] in Respiratory specimen by Rapid immunoassay – positive, ever
LOINC 94763-0: SARS-CoV-2 (COVID-19) [Presence] in Unspecified specimen by Organism specific culture – positive, ever
LOINC 96603-6: SARS-CoV-2 (COVID-19) S protein RBD neutralizing antibody [Presence] in Serum or Plasma by Immunoassay – positive, ever
LOINC 96119-3: SARS-CoV-2 (COVID-19) Ag [Presence] in Upper respiratory specimen by Immunoassay – positive, ever

ORI Cohort: Inclusion and Exclusion Criteria Codes

Included if any of the codes below from January 1, 2020 to December 31, 2022:

J00-J06: Acute upper respiratory infections – 10-19 years
J09-J18: Influenza and pneumonia – 10-19 years
J20-J22: Other acute lower respiratory infections – 10-19 years

Excluded if any of the code below at least 1 day before the first instance of the code above:

E11: Type 2 diabetes mellitus
9037 (TNX curated): Hemoglobin A1c/Hemoglobin total in blood ≥ 6.5%

Excluded if any code below occurred anytime:

E10: Type 1 diabetes

Excluded if any code below on or before June 30, 2023:

B97.29: Other coronavirus as the cause of diseases classified elsewhere
J12.82: Pneumonia due to COVID-19
J12.81: Pneumonia due to SARS-associated coronavirus
B34.2: Coronavirus infection, unspecified
U07.1: COVID-19
U07.2: COVID-19, virus not identified (WHO)
9088 (TNX curated): SARS coronavirus 2 and related RNA [Presence] – positive, ever
LOINC 94558-4: SARS-CoV-2 (COVID-19) Ag [Presence] in Respiratory specimen by Rapid immunoassay – positive, ever
LOINC 97097-0: SARS-CoV-2 (COVID-19) Ag [Presence] in Upper respiratory specimen by Rapid immunoassay – positive, ever
LOINC 95209-3: SARS coronavirus + SARS coronavirus 2 Ag [Presence] in Respiratory specimen by Rapid immunoassay – positive, ever
LOINC 94763-0: SARS-CoV-2 (COVID-19) [Presence] in Unspecified specimen by Organism specific culture – positive, ever
LOINC 96603-6: SARS-CoV-2 (COVID-19) S protein RBD neutralizing antibody [Presence] in Serum or Plasma by Immunoassay – positive, ever
LOINC 96119-3: SARS-CoV-2 (COVID-19) Ag [Presence] in Upper respiratory specimen by Immunoassay – positive, ever

© 2024 Miller MG et al. *JAMA Network Open*.

Excluded if any code below on or before December 31, 2020:

9089 (TNX curated): SARS coronavirus 2 IgG IgM Ab [Presence] in Serum or Plasma – positive, ever
LOINC 94505-5: SARS-CoV-2 (COVID-19) IgG Ab [Units/volume] in Serum or Plasma by Immunoassay ≥0.1 [arb'U]/ml, ever
LOINC 94506-3: SARS-CoV-2 (COVID-19) IgM Ab [Units/volume] in Serum or Plasma by Immunoassay ≥0.1 [arb'U]/ml, ever
LOINC 94562-6: SARS-CoV-2 (COVID-19) IgA Ab [Presence] in Serum or Plasma by Immunoassay – positive, ever
LOINC 94762-2: SARS-CoV-2 (COVID-19) Ab [Presence] in Serum or Plasma by Immunoassay – positive, ever
LOINC 94769-7: SARS-CoV-2 (COVID-19) Ab [Units/volume] in Serum or Plasma by Immunoassay ≥0.1 [IU]/ml, ever

Patients with Overweight or Obesity

Included if either code occurred within 5 years of the COVID-19 or ORI event

Z68.54: Body mass index [BMI] pediatric, greater than or equal to 95th percentile for age
Z68.53: Body mass index [BMI] pediatric, 85th percentile to less than 95th percentile for age

Hospitalized Patients

Included if an "Inpatient Encounter" visit documented from 1 week prior to 1 month after COVID-19 or ORI event

Prediabetic Patients

Included if these codes all occurred at least one day before the first instance of the COVID-19 or ORI event

LOINC 1558-6: Fasting glucose [Mass/volume] in Serum or Plasma ≥100 mg/dL
TNX curated 9004: Triglyceride [Mass/volume] in Serum, Plasma or Blood ≥150 mg/dL
TNX curated 9085: Blood Pressure, Systolic ≥130 mmHg
or
TNX curated 9086: Blood Pressure, Diastolic ≥85 mmHg

Nonviral Comparison Cohort: Inclusion and Exclusion Criteria Codes

Included if any of the codes below from January 1, 2020 to December 31, 2022:

L70.0: Acne Vulgaris – 10-19 years
S42: Fracture of shoulder and upper arm – 10-19 years
S52: Fracture of forearm – 10-19 years
Z01.0: Encounter for examination of eyes and vision – 10-19 years
Z46.0: Encounter for fitting and adjustment of spectacles and contact lenses – 10-19 years

Exclusion criteria were the same as those for the ORI Cohort

TNX curated 9088: composite code created by TriNetX and includes RNA detection tests for SARS-CoV-2, which includes any of the codes below:

LOINC 94308-4:
SARS-CoV-2 (COVID-19) N gene [Presence] in Specimen by Nucleic acid amplification using CDC primer-probe set N2
LOINC 94309-2: SARS-CoV-2 (COVID-19) RNA [Presence] in Specimen by NAA with probe detection
LOINC 94310-0: SARS-related coronavirus N gene [Presence] in Specimen by Nucleic acid amplification using CDC primer-probe set N3
LOINC 94314-2: SARS-CoV-2 (COVID-19) RdRp gene [Presence] in Specimen by NAA with probe detection

© 2024 Miller MG et al. *JAMA Network Open*.

LOINC 94315-9: SARS-related coronavirus E gene [Presence] in Specimen by NAA with probe detection
LOINC 94316-7: SARS-CoV-2 (COVID-19) N gene [Presence] in Specimen by NAA with probe detection
LOINC 94500-6: SARS-CoV-2 (COVID-19) RNA [Presence] in Respiratory specimen by NAA with probe detection
LOINC 94502-2: SARS-related coronavirus RNA [Presence] in Respiratory specimen by NAA with probe detection
LOINC 94533-7: SARS-CoV-2 (COVID-19) N gene [Presence] in Respiratory specimen by NAA with probe detection
LOINC 94534-5: SARS-CoV-2 (COVID-19) RdRp gene [Presence] in Respiratory specimen by NAA with probe detection
LOINC 94559-2: SARS-CoV-2 (COVID-19) ORF1ab region [Presence] in Respiratory specimen by NAA with probe detection
LOINC 94565-9: SARS-CoV-2 (COVID-19) RNA [Presence] in Nasopharynx by NAA with non-probe detection
LOINC 94639-2: SARS-CoV-2 (COVID-19) ORF1ab region [Presence] in Specimen by NAA with probe detection
LOINC 94647-5: SARS-related coronavirus RNA [Presence] in Specimen by NAA with probe detection
LOINC 94758-0: SARS-related coronavirus E gene [Presence] in Respiratory specimen by NAA with probe detection
LOINC 94759-8: SARS-CoV-2 (COVID-19) RNA [Presence] in Nasopharynx by NAA with probe detection
LOINC 94760-6: SARS-CoV-2 (COVID-19) N gene [Presence] in Nasopharynx by NAA with probe detection
LOINC 94845-5: SARS-CoV-2 (COVID-19) RNA [Presence] in Saliva (oral fluid) by NAA with probe detection
LOINC 95406-5: SARS-CoV-2 (COVID-19) RNA [Presence] in Nose by NAA with probe detection
LOINC 95409-9: SARS-CoV-2 (COVID-19) N gene [Presence] in Nose by NAA with probe detection
LOINC 95608-6: SARS-CoV-2 (COVID-19) RNA [Presence] in Respiratory specimen by NAA with non-probe detection
LOINC 96123-5: SARS-CoV-2 (COVID-19) RdRp gene [Presence] in Upper respiratory specimen by NAA with probe detection
LOINC 96763-8: SARS-CoV-2 (COVID-19) E gene [Presence] in Respiratory specimen by NAA with probe detection

TNX curated 9037: composite code for Hemoglobin A1c/Hemoglobin total in blood, which includes any of the codes below:
LOINC 17855-8: Hemoglobin A1c/Hemoglobin total in Blood by calculation (191,838)
LOINC 1785606: Hemoglobin A1c/Hemoglobin total in Blood by HPCL (1,702,618)
LOINC 4548-4: Hemoglobin A1c/Hemoglobin total in Blood (12,138,581)
LOINC 4549-2: Hemoglobin A1c/Hemoglobin total in Blood by Electrophoresis (4,884)

TNX curated 9089: composite code for SARS coronavirus 2 IgG IgM Ab [Presence] in Serum or Plasma, which includes any of the codes below:
LOINC 94507-1: SARS-CoV-2 (COVID-19) IgG Ab [Presence] in Serum, Plasma or Blood by Rapid immunoassay (98,829)
LOINC 94508-9: SARS-CoV-2 (COVID-19) IgM Ab [Presence] in Serum, Plasma or Blood by Rapid immunoassay (1,412)
LOINC 94547-7: SARS-CoV-2 (COVID-19) IgG+IgM Ab [Presence] in Serum or Plasma by Immunoassay (13,067)
LOINC 94563-4: SARS-CoV-2 (COVID-19) IgG Ab [Presence] in Serum or Plasma by Immunoassay (254,033)
LOINC 94564-2: SARS-CoV-2 (COVID-19) IgM Ab [Presence] in Serum or Plasma by Immunoassay (8,293)
LOINC 94761-4: SARS-CoV-2 (COVID-19) IgG Ab [Presence] in DBS by Immunoassay (10)
LOINC 99596-9: SARS-CoV-2 (COVID-19) N protein IgG Ab [Presence] in Serum or Plasma by Immunoassay (4,432)

© 2024 Miller MG et al. *JAMA Network Open*.

LOINC 99597-7: SARS-CoV-2 (COVID-19) S protein IgG Ab [Presence] in Serum or Plasma by Immunoassay (559)

TNX curated 9004: a composite code for Triglyceride [Mass/volume] in Serum, Plasma or Blood, which includes any of the codes below:

LOINC 12951-0: Triglyceride [Mass/volume] in Serum or Plasma by calculation
LOINC 2571-8: Triglyceride [Mass/volume] in Serum or Plasma
LOINC 3043-7: Triglyceride [Mass/volume] in Blood

TNX curated 9085: a composite code for Blood Pressure, Systolic, which includes any of the codes below:

LOINC 8480-6: Blood Pressure Systolic
LOINC 76215-3: Invasive Systolic blood pressure
LOINC 76534-7: Systolic blood pressure by Noninvasive
LOINC 87739-9: Systolic blood pressure—W exercise
LOINC 87741-5: Systolic blood pressure—post exercise
LOINC 8459-0: Systolic blood pressure—sitting
LOINC 8460-8: Systolic blood pressure—standing
LOINC 8461-6: Systolic blood pressure—supine

TNX curated 9086: a composite code for Blood Pressure, Diastolic, which includes any of the codes below:

LOINC 8462-4: Blood Pressure Diastolic
LOINC 76213-8: Invasive Diastolic blood pressure
LOINC 76535-4: Diastolic blood pressure by Noninvasive
LOINC 87740-7: Diastolic blood pressure—W exercise
LOINC 87736-5: Diastolic blood pressure—post exercise
LOINC 8453-3: Diastolic blood pressure—sitting
LOINC 8454-1: Diastolic blood pressure—standing
LOINC 8455-8: Diastolic blood pressure--supine

Baseline Characteristics Criteria Codes for Matching
Documentation in the patient record anytime up to 1 day prior to the index event

Age: Current Age
AI: Age at Index
F: Female
M: Male
UN: Unknown Gender
2135-2: Hispanic or Latino
2186-5: Not Hispanic or Latino
UN: Unknown Ethnicity
1002-5: American Indian or Alaska Native
2028-9: Asian
2054-5: Black or African American
2086-8: Native Hawaiian or Other Pacific Islander
2106-3: White
2131-1: Other Race
9000 (TNX Curated): Cholesterol [Mass/volume] in Serum or Plasma (≥200 mg/dL)
Z68.5: Body mass index [BMI] pediatric
Z68.54: Body mass index [BMI] pediatric, greater than or equal to 95th percentile for age
Z68.53: Body mass index [BMI] pediatric, 85th percentile to less than 95th percentile for age
I10: Essential (primary) hypertension
Z83.3: Family history of diabetes mellitus
E78.0: Pure hypercholesterolemia
Z79.52: Long term (current) use of systemic steroids
P07.1: Other low birth weight newborn

© 2024 Miller MG et al. *JAMA Network Open*.

L40: Psoriasis
P07.0: Extremely low birth weight newborn
E28.2: Polycystic ovarian syndrome
E06.3: Autoimmune thyroiditis
K90.0: Celiac disease
K50: Crohn's disease [regional enteritis]
I05-I09: Chronic rheumatic heart diseases
I15.8: Other secondary hypertension
K51: Ulcerative colitis
E05: Thyrotoxicosis [hyperthyroidism]
M32: Systemic lupus erythematosus (SLE)
D69.3: Immune thrombocytopenic purpura
M06.9: Rheumatoid arthritis, unspecified
K75.4: Autoimmune hepatitis
G35: Multiple sclerosis
M35.0: Sjögren syndrome
G70.0: Myasthenia gravis
M45: Ankylosing spondylitis
D86: Sarcoidosis
M34: Systemic sclerosis [scleroderma]
P70.0: Syndrome of infant of mother with gestational diabetes

© 2024 Miller MG et al. *JAMA Network Open*.

eTable 1. Baseline Characteristics of Pediatric Patient Cohorts With Overweight or Obesity Before and After Propensity Score Matching

eTable 1. Baseline Characteristics of Overweight or Obese Pediatric Patient Cohorts Before and After Propensity Score Matching

Characteristic[a]	Cohort before matching, No (%)[b]			Cohort after matching, No (%)[b]		
	COVID-19 (n = 16 538)	ORI (n = 43 151)	SMD[c]	COVID-19 (n = 16 496)	ORI (n = 16 496)	SMD[c]
Age when data accessed, mean (SD), y	17.3 (2.9)	16.6 (3.1)	0.21	17.3 (2.9)	17.2 (2.9)	0.007
Age at index, mean (SD), y[d]	15.0 (2.7)	14.2 (2.9)	0.26	15.0 (2.7)	15.0 (2.7)	0.002
Sex						
Female	8 475 (51.2)	21 924 (50.8)	0.009	8 449 (51.2)	8 445 (51.2)	5E-04
Male	7 857 (47.5)	20 297 (47.0)	0.001	7 841 (47.5)	7 843 (47.5)	2E-04
Unknown Gender	206 (1.2)	940 (2.2)	0.07	206 (1.2)	208 (1.3)	0.001
Ethnicity[e]						
Hispanic or Latinx	3 885 (23.5)	8 433 (19.5)	0.1	3 863 (23.4)	3 812 (23.1)	0.007
Not Hispanic or Latinx	10 402 (62.9)	28 465 (66.0)	0.06	10 385 (63.0)	10 459 (63.4)	0.009
Unknown	2 251 (13.6)	6 253 (14.5)	0.03	2 248 (13.6)	2 225 (13.5)	0.004
Race[e]						
American Indian or Alaska Native	109 (0.7)	260 (0.6)	0.007	109 (0.7)	97 (0.6)	0.009
Asian	462 (2.8)	1 186 (2.7)	0.003	462 (2.8)	468 (2.8)	0.002
Black or African American	3 526 (21.3)	7 054 (16.3)	0.13	3 512 (21.3)	3 622 (22.0)	0.02
Native Hawaiian or Other Pacific Islander	86 (0.5)	207 (0.5)	0.006	86 (0.5)	74 (0.4)	0.01
White	8 824 (53.4)	24 939 (57.8)	0.09	8 805 (53.4)	8 719 (52.9)	0.01
Other Race[f]	1 222 (7.4)	3 059 (7.1)	0.01	1 216 (7.4)	1 236 (7.5)	0.005
Cholesterol measured	7 943 (48.0)	16 608 (38.5)	0.003	7 922 (48.0)	7 166 (43.4)	0.01
Cholesterol, ≥ 200 mg/dL	978 (5.9)	1 979 (4.6)	0.06	970 (5.9)	970 (5.9)	0
BMI documented	16 538 (100.0)	43 151 (100.0)		16 496 (100.0)	16 496 (100.0)	
BMI, pediatric, ≥95th percentile for age	11 418 (69.0)	27 065 (62.7)	0.13	11 384 (69.0)	10 677 (64.7)	0.09
BMI, pediatric, 85th < 95th percentile for age	7 048 (42.6)	21 225 (49.2)	0.13	7 032 (42.6)	7 844 (47.6)	0.1
Primary hypertension	964 (5.8)	1 451 (3.4)	0.12	933 (5.7)	911 (5.5)	0.006
Family history of diabetes mellitus	943 (5.7)	1 758 (4.1)	0.08	928 (5.6)	903 (5.5)	0.007
Pure hypercholesterolemia	557 (3.4)	1 293 (3.0)	0.02	554 (3.4)	516 (3.1)	0.01
Long term use of systemic steroids	206 (1.2)	285 (0.7)	0.06	185 (1.1)	168 (1.0)	0.01
Other low birth weight newborn	129 (0.8)	278 (0.6)	0.02	125 (0.8)	92 (0.6)	0.02
Psoriasis	101 (0.6)	250 (0.6)	0.004	100 (0.6)	90 (0.5)	0.008
Extremely low birth weight newborn	88 (0.5)	194 (0.5)	0.01	86 (0.5)	60 (0.4)	0.02

© 2024 Miller MG et al. *JAMA Network Open*.

Polycystic ovarian syndrome	225 (1.4)	403 (0.9)	0.04	224 (1.4)	217 (1.3)	0.004
Autoimmune thyroiditis	122 (0.7)	274 (0.6)	0.01	120 (0.7)	91 (0.6)	0.02
Celiac disease	53 (0.3)	114 (0.3)	0.01	52 (0.3)	38 (0.2)	0.02
Crohn's disease	44 (0.3)	72 (0.2)	0.02	44 (0.3)	44 (0.3)	0
Chronic rheumatic heart diseases	66 (0.4)	77 (0.2)	0.04	56 (0.3)	52 (0.3)	0.004
Secondary hypertension	120 (0.7)	112 (0.3)	0.07	97 (0.6)	88 (0.5)	0.007
Ulcerative colitis	36 (0.2)	45 (0.1)	0.03	36 (0.2)	31 (0.2)	0.007
Hyperthyroidism	49 (0.3)	82 (0.2)	0.02	48 (0.3)	30 (0.2)	0.02
Systemic lupus erythematosus	45 (0.3)	37 (0.1)	0.04	33 (0.2)	27 (0.2)	0.009
Immune thrombocytopenic purpura	29 (0.2)	41 (0.1)	0.02	25 (0.2)	24 (0.1)	0.002
Rheumatoid arthritis	11 (0.07)	11 (0.03)	0.02	10[g] (0.06)	10[g] (0.06)	0
Autoimmune hepatitis	13 (0.08)	10[g] (0.02)	0.01	10[g] (0.06)	10[g] (0.06)	0
Multiple sclerosis	10[g] (0.06)	10[g] (0.02)	0.03	10[g] (0.06)	10[g] (0.06)	0
Sjögren syndrome	10[g] (0.06)	10[g] (0.02)	0.03	10[g] (0.06)	10[g] (0.06)	0
Myasthenia gravis	10[g] (0.06)	10[g] (0.02)	0.03	10[g] (0.06)	10[g] (0.06)	0
Ankylosing spondylitis	10[g] (0.06)	10[g] (0.02)	0.03	10[g] (0.06)	10[g] (0.06)	0
Sarcoidosis	10[g] (0.06)	10[g] (0.02)	0.03	10[g] (0.06)	10[g] (0.06)	0
Systemic sclerosis	10[g] (0.06)	10[g] (0.02)	0.03	10[g] (0.06)	10[g] (0.06)	0
Infant of mother with gestational diabetes	10[g] (0.06)	10[g] (0.02)	0.03	10[g] (0.06)	10[g] (0.06)	0

Abbreviaton: ORI, other respiratory infections; SMD, standardized mean difference; BMI, body mass index

[a] Patient characteristics on the day of index (infection)
[b] Data are presented as number (percentage) of patients unless otherwise indicated.
[c] An SMD greater than 0.1 is a threshold recommended for declaring imbalance.
[d] Index event was diagnosis of COVID-19 or other respiratory infection.
[e] Race and ethnicity were self-reported or provider observed and were included because they are reported to be associated with COVID-19 diagnosis and severity.
[f] Includes a patient if they identify as multi-racial (more than one race specified) or if the patient belongs to a race that is not listed in the aforementioned options.
[g] The TriNetX platform reports counts of 1-10 as 10 for addition privacy protection.

SI conversion factors: To convert cholesterol to mmol/L, multiply values by 0.0259.

© 2024 Miller MG et al. *JAMA Network Open*.

eTable 2. Baseline Characteristics of Inpatient Pediatric Patient Cohorts Before and After Propensity Score Matching

**eTable 2. Baseline Characteristics of Inpatient Pediatric Patient Cohorts Before and After Propensity Score Matching**						
	Cohort before matching, No (%)[b]			Cohort after matching, No (%)[b]		
Characteristic[a]	COVID-19 (n = 14 094)	ORI (n = 22 599)	SMD	COVID-19 (n = 13 653)	ORI (n = 13 653)	SMD
Age when data accessed, mean (SD), y	17.7 (2.9)	17.4 (3.1)	0.10	17.7 (2.9)	17.4 (3.0)	0.08
Age at index, mean (SD), y[d]	15.4 (2.8)	14.8 (2.8)	0.19	15.3 (2.8)	15.1 (2.8)	0.04
Sex						
Female	7 928 (56.3)	11 791 (52.2)	0.08	7 577 (55.5)	7 360 (53.9)	0.03
Male	6 128 (43.5)	10 762 (47.6)	0.08	6 040 (44.2)	6 258 (45.8)	0.03
Unknown Gender	38 (0.3)	46 (0.2)	0.01	36 (0.3)	35 (0.3)	0.003
Ethnicity[e]						
Hispanic or Latinx	2 760 (19.6)	3 350 (14.8)	0.13	2 583 (18.9)	2 481 (18.2)	0.02
Not Hispanic or Latinx	9 581 (68.0)	17 271 (76.4)	0.19	9 439 (69.1)	9 636 (70.6)	0.03
Unknown	1 753 (12.4)	1 978 (8.8)	0.12	1 631 (11.9)	1 536 (11.3)	0.02
Race[e]						
American Indian or Alaska Native	107 (0.8)	145 (0.6)	0.01	103 (0.8)	104 (0.8)	8E-04
Asian	322 (2.3)	1433 (1.9)	0.03	308 (2.3)	295 (2.2)	0.006
Black or African American	3 201 (22.7)	4 343 (19.2)	0.09	3 089 (22.6)	3 106 (22.8)	0.003
Native Hawaiian or Other Pacific Islander	62 (0.4)	57 (0.3)	0.03	58 (0.4)	44 (0.3)	0.02
White	7 368 (52.3)	14 508 (64.2)	0.24	7 295 (53.4)	7 505 (55.0)	0.03
Other Race[f]	1 096 (7.8)	1 143 (5.1)	0.11	1 013 (7.4)	931 (6.8)	0.02
Cholesterol measured	2 573 (18.3)	5 269 (23.3)	0.002	2 509 (18.4)	2 990 (21.9)	0.003
Cholesterol, ≥ 200 mg/dL	397 (2.8)	691 (3.1)	0.01	383 (2.8)	383 (2.8)	0
BMI documented	3 117 (22.1)	8 326 (36.8)	0.33	3 108 (22.8)	3 217 (23.6)	0.02
BMI, pediatric, ≥95th percentile for age	1 476 (10.5)	3 090 (13.7)	0.1	1 469 (10.8)	1 560 (11.4)	0.02
BMI, pediatric, 85th < 95th percentile for age	664 (4.7)	1 972 (8.7)	0.16	663 (4.9)	657 (4.8)	0.002
Primary hypertension	1 176 (8.3)	1 676 (7.4)	0.03	1 140 (8.4)	1 146 (8.4)	0.002
Family history of diabetes mellitus	542 (3.8)	815 (3.6)	0.01	523 (3.8)	496 (3.6)	0.01
Pure hypercholesterolemia	148 (1.1)	293 (1.3)	0.02	148 (1.1)	135 (1.0)	0.009
Long term use of systemic steroids	381 (2.7)	601 (2.7)	0.003	369 (2.7)	368 (2.7)	5E-04
Other low birth weight newborn	102 (0.7)	232 (1.0)	0.03	101 (0.7)	102 (0.7)	9E-04
Psoriasis	72 (0.5)	143 (0.6)	0.02	72 (0.5)	67 (0.5)	0.005
Extremely low birth weight newborn	65 (0.4)	149 (0.7)	0.04	56 (0.4)	58 (0.4)	0.002
Polycystic ovarian syndrome	73 (0.5)	91 (0.4)	0.02	70 (0.5)	65 (0.5)	0.005
Autoimmune thyroiditis	72 (0.5)	150 (0.7)	0.02	72 (0.5)	74 (0.4)	0.002

© 2024 Miller MG et al. *JAMA Network Open*.

Celiac disease	46 (0.3)	121 (0.5)	0.03	46 (0.3)	43 (0.3)	0.004
Crohn's disease	139 (1.0)	232 (1.0)	0.004	137 (1.0)	123 (0.9)	0.01
Chronic rheumatic heart diseases	173 (1.2)	255 (1.1)	0.009	165 (1.2)	169 (1.2)	0.003
Secondary hypertension	220 (1.6)	305 (1.4)	0.02	216 (1.6)	206 (1.5)	0.006
Ulcerative colitis	108 (0.8)	153 (0.7)	0.01	102 (0.7)	95 (0.7)	0.006
Hyperthyroidism	64 (0.5)	90 (0.4)	0.009	60 (0.4)	60 (0.4)	0
Systemic lupus erythematosus	89 (0.6)	128 (0.6)	0.008	82 (0.6)	76 (0.6)	0.006
Immune thrombocytopenic purpura	61 (0.4)	72 (0.3)	0.02	56 (0.4)	53 (0.4)	0.003
Rheumatoid arthritis	17 (0.1)	25 (0.1)	0.003	15 (0.1)	12 (0.09)	0.007
Autoimmune hepatitis	30 (0.2)	24 (0.1)	0.03	24 (0.2)	22 (0.2)	0.004
Multiple sclerosis	26 (0.2)	18 (0.08)	0.03	21 (0.15)	18 (0.13)	0.006
Sjögren syndrome	10[g] (0.07)	12 (0.05)	0.007	10[g] (0.07)	10[g] (0.07)	0
Myasthenia gravis	15 (0.1)	15 (0.07)	0.01	15 (0.1)	14 (0.1)	0.002
Ankylosing spondylitis	0 (0)	10[g] (0.04)	0.03	0 (0)	0 (0)	
Sarcoidosis	10[g] (0.07)	10[g] (0.04)	0.01	10[g] (0.07)	10[g] (0.07)	0
Systemic sclerosis	10[g] (0.07)	14 (0.06)	0.003	10[g] (0.07)	10[g] (0.07)	0
Infant of mother with gestational diabetes	10[g] (0.07)	0 (0)	0.04	10[g] (0.07)	0 (0)	0.04

Abbreviaton: ORI, other respiratory infections; SMD, standardized mean difference; BMI, body mass index

[a] Patient characteristics on the day of index (infection)
[b] Data are presented as number (percentage) of patients unless otherwise indicated.
[c] An SMD greater than 0.1 is a threshold recommended for declaring imbalance.
[d] Index event was diagnosis of COVID-19 or other respiratory infection.
[e] Race and ethnicity were self-reported or provider observed and were included because they are reported to be associated with COVID-19 diagnosis and severity.
[f] Includes a patient if they identify as multi-racial (more than one race specified) or if the patient belongs to a race that is not listed in the aforementioned options.
[g] The TriNetX platform reports counts of 1-10 as 10 for addition privacy protection.

SI conversion factors: To convert cholesterol to mmol/L, multiply values by 0.0259.

© 2024 Miller MG et al. *JAMA Network Open*.

eTable 3. Comparison of Risk of New Diagnoses of T2D in Patients Aged 10 to 19 Years by Sex (Male vs Female) at 1, 3, and 6 Months From the Same Day of COVID-19 Diagnosis

**eTable 3.** Comparison of Risk of New Diagnoses of T2DM in Patients ages 10-19 years by Sex (Male vs. Female) at 1 month, 3 months, and 6 months from the Same Day of COVID-19 Diagnosis			
	**Patients with New Type 2 Diabetes,[a] No (%)**		
**Time since index infection, mo**	**Male Cohort with COVID-19**	**Female Cohort with COVID-19**	**RR (95% CI)**
	**(n = 147 598)**	**(n = 147 598)**	
1	107 (0.07)	115 (0.08)	0.98 (0.72-1.21)
3	131 (0.09)	143 (0.10)	0.92 (0.72-1.16)
6	166 (0.12)	183 (0.13)	0.91 (0.74-1.12)

Abbreviations: ORI, other respiratory infection; RR, relative risk

[a]Patients with new documentation of T2DM by ICD-10 code

© 2024 Miller MG et al. *JAMA Network Open*.

eTable 4. Baseline Characteristics of Pediatric Patient Cohorts With Prediabetes Before and After Propensity Score Matching

**eTable 4. Baseline Characteristics of Prediabetic Pediatric Patient Cohorts Before and After Propensity Score Matching**						
	Cohort before matching, No (%)[b]			Cohort after matching, No (%)[b]		
Characteristic[a]	COVID-19 (n = 5 024)	ORI (n = 8 626)	SMD[c]	COVID-19 (n = 4 996)	ORI (n = 4 996)	SMD[c]
Age when data accessed, mean (SD), y	18.2 (2.7)	17.9 (2.9)	0.1	18.2 (2.7)	18.2 (2.8)	0.02
Age at index, mean (SD), y[d]	15.2 (2.5)	14.8 (2.7)	0.18	15.2 (2.7)	15.2 (2.6)	0.008
Sex						
Female	2 629 (52.3)	4 616 (53.5)	0.02	2 616 (52.4)	2 624 (52.5)	0.003
Male	2 369 (47.2)	3970 (46.0)	0.02	2 355 (47.1)	2 345 (46.9)	0.004
Unknown Gender	26 (0.5)	40 (0.5)	0.008	25 (0.5)	27 (0.5)	0.006
Ethnicity[e]						
Hispanic or Latinx	1 419 (28.2)	2 116 (24.5)	0.08	1 401 (28.0)	1 362 (27.3)	0.02
Not Hispanic or Latinx	3 018 (60.1)	5 519 (64.0)	0.08	3 009 (60.2)	3 045 (61.0)	0.01
Unknown	587 (11.7)	991 (11.5)	0.006	586 (11.7)	589 (11.8)	0.002
Race[e]						
American Indian or Alaska Native	30 (0.6)	61 (0.7)	0.01	30 (0.6)	24 (0.5)	0.02
Asian	113 (2.3)	246 (2.9)	0.04	113 (2.3)	87 (1.7)	0.04
Black or African American	1 798 (35.8)	2 906 (33.7)	0.04	1 791 (35.9)	1 802 (36.1)	0.005
Native Hawaiian or Other Pacific Islander	26 (0.5)	38 (0.4)	0.01	26 (0.5)	24 (0.5)	0.006
White	1 756 (35.0)	3 236 (37.5)	0.05	1 744 (34.9)	1 770 (35.4)	0.01
Other Race[f]	540 (10.8)	897 (10.4)	0.01	539 (10.8)	538 (10.8)	6E-04
Cholesterol measured	4 397 (15.1)	7 504 (14.3)	0.01	4 372 (87.5)	4 355 (87.2)	0.003
Cholesterol, ≥ 200 mg/dL	726 (1.6)	1 151 (13.3)	0.03	719 (14.4)	669 (13.4)	0.03
BMI documented	3 140 (62.5)	5 485 (63.6)	0.02	3 122 (62.5)	3 141 (62.9)	0.008
BMI, pediatric, ≥ 95th percentile for age	2 758 (54.9)	4 744 (55.0)	0.002	2 741 (54.9)	2 783 (55.7)	0.02
BMI, pediatric, 85th < 95th percentile for age	640 (12.7)	1 100 (12.8)	4E-04	635 (12.7)	597 (12.0)	0.02
Primary hypertension	492 (9.8)	711 (8.2)	0.05	481 (9.6)	425 (8.5)	0.04
Family history of diabetes mellitus	471 (9.4)	803 (9.3)	0.002	467 (9.4)	425 (8.5)	0.03
Pure hypercholesterolemia	357 (7.1)	595 (6.9)	0.008	356 (7.1)	321 (6.4)	0.03
Long term use of systemic steroids	79 (1.6)	98 (1.1)	0.04	74 (1.5)	60 (1.2)	0.02
Other low birth weight newborn	49 (1.0)	83 (1.0)	0.001	49 (1.0)	42 (0.8)	0.01
Psoriasis	31 (0.6)	72 (0.8)	0.03	31 (0.6)	24 (0.5)	0.02
Extremely low birth weight newborn	34 (0.7)	60 (0.7)	0.002	34 (0.7)	32 (0.7)	0.005
Polycystic ovarian syndrome	173 (3.4)	265 (3.1)	0.02	171 (3.4)	166 (3.3)	0.006
Autoimmune thyroiditis	67 (1.3)	105 (1.2)	0.01	66 (1.3)	61 (1.2)	0.009
Celiac disease	15 (0.3)	36 (0.4)	0.02	15 (0.3)	20 (0.4)	0.02
Crohn's disease	21 (0.4)	29 (0.3)	0.01	20 (0.4)	16 (0.3)	0.01

© 2024 Miller MG et al. *JAMA Network Open*.

Chronic rheumatic heart diseases	21 (0.4)	42 (0.5)	0.01	21 (0.4)	17 (0.3)	0.01
Secondary hypertension	55 (1.1)	62 (0.7)	0.04	47 (1.0)	33 (0.7)	0.03
Ulcerative colitis	16 (0.3)	16 (0.2)	0.03	12 (0.2)	12 (0.2)	0
Hyperthyroidism	17 (0.3)	29 (0.3)	3E-04	17 (0.3)	20 (0.4)	0.01
Systemic lupus erythematosus	10[g] (0.2)	14 (0.2)	0.009	10[g] (0.2)	10[g] (0.2)	0
Immune thrombocytopenic purpura	12 (0.2)	10[g] (0.1)	0.03	10[g] (0.2)	10[g] (0.2)	0
Rheumatoid arthritis	10[g] (0.2)	10[g] (0.1)	0.02	10[g] (0.2)	10[g] (0.2)	0
Autoimmune hepatitis	10[g] (0.2)	10[g] (0.1)	0.02	10[g] (0.2)	0 (0.00)	0.06
Multiple sclerosis	10[g] (0.2)	10[g] (0.1)	0.02	10[g] (0.2)	10[g] (0.2)	0
Sjögren syndrome	10[g] (0.2)	10[g] (0.1)	0.02	10[g] (0.2)	10[g] (0.2)	0
Myasthenia gravis	10[g] (0.2)	10[g] (0.1)	0.02	10[g] (0.2)	10[g] (0.2)	0
Ankylosing spondylitis	10[g] (0.2)	10[g] (0.1)	0.02	10[g] (0.2)	10[g] (0.2)	0
Sarcoidosis	10[g] (0.2)	10[g] (0.1)	0.02	10[g] (0.2)	10[g] (0.2)	0
Systemic sclerosis	10[g] (0.2)	10[g] (0.1)	0.02	10[g] (0.2)	0 (0.00)	0.06
Infant of mother with gestational diabetes	10[g] (0.2)	10[g] (0.1)	0.02	10[g] (0.2)	10[g] (0.2)	0

Abbreviation: ORI, other respiratory infections; SMD, standardized mean difference; BMI, body mass index

[a] Patient characteristics up to 1 day prior to index infection
[b] Data are presented as number (percentage) of patients unless otherwise indicated.
[c] An SMD greater than 0.1 is a threshold recommended for declaring imbalance.
[d] Index event was diagnosis of COVID-19 or other respiratory infection.
[e] Race and ethnicity were self-reported or provider observed and were included because they are reported to be associated with COVID-19 diagnosis and severity.
[f] Includes a patient if they identify as multi-racial (more than one race specified) or if the patient belongs to a race that is not listed in the aforementioned options.
[g] The TriNetX platform reports counts of 1-10 as 10 for addition privacy protection.

SI conversion factors: To convert cholesterol to mmol/L, multiply values by 0.0259.

© 2024 Miller MG et al. *JAMA Network Open*.

eTable 5. Comparison of Risk of New Diagnoses of T2D in Patients Aged 10 to 19 Years With Prediabetes at 1, 3, and 6 Months From the Same Day of Respiratory Illness Diagnosis

**eTable 5.** Comparison of Risk of New Diagnoses of T2DM in Patients ages 10-19 years with Prediabetes at 1 month, 3 months, and 6 months from the Same Day of Respiratory Illness Diagnosis			
	**Patients with New Type 2 Diabetes,[a] No (%)**		
**Time since index infection, mo; number in matched cohorts**	**COVID-19 (n = 4 996)**	**ORI (n = 4 996)**	**RR (95% CI)**
1	26 (0.52)	14 (0.28)	1.86 (0.97-3.55)
3	35 (0.70)	20 (0.4)	1.75 (1.01-3.03)
6	49 (0.98)	34 (0.68)	1.44 (0.93-2.23)

Abbreviations: ORI, other respiratory infection; RR, relative risk

[a]Patients with new documentation of T2DM by ICD-10 code

© 2024 Miller MG et al. *JAMA Network Open*.

eTable 6. Baseline Characteristics of Pediatric Patient Cohorts With Viral and Nonviral Diagnoses Before and After Propensity Score Matching

eTable 6. Baseline Characteristics of Viral and Nonviral Pediatric Patient Cohorts Before and After Propensity Score Matching						
	Cohort before matching, No (%)[b]			Cohort after matching, No (%)[b]		
Characteristic[a]	COVID-19 (n = 369 515	Nonviral Encounter (n = 373 886)	SMD[c]	COVID-19 (n = 302 119)	Nonviral Encounter (n = 302 119)	SMD[c]
Age when data accessed, mean (SD), y	17.9 (3.1)	17.3 (2.8)	0.19	17.5 (3.1)	17.3 (2.9)	0.07
Age at index, mean (SD), y[d]	14.9 (2.9)	14.1 (2.7)	0.28	14.5 (2.9)	14.3 (2.7)	0.05
Sex						
Female	190 074 (51.4)	170 255 (45.5)	0.1	145 276 48.1)	141 310 (46.8)	0.03
Male	171 388 (46.4)	189 864 (50.8)	0.09	148 950 (49.3)	152 960 (50.6)	0.03
Unknown Gender	8 053 (2.2)	13 767 (3.7)	0.09	7 893 (2.6)	7 849 (2.6)	9E-04
Ethnicity[e]						
Hispanic or Latinx	65 755 (17.8)	60 669 (16.2)	0.04	51 966 (17.2)	52 565 (17.4)	0.005
Not Hispanic or Latinx	240 244 (65.0)	249 025 (66.6)	0.03	199 004 (65.9)	198 423 (65.7)	0.004
Unknown	63 516 (17.2)	64 192 (17.2)	5E-04	51 149 (16.9)	51 131 (16.9)	2E-04
Race[e]						
American Indian or Alaska Native	1 308 (0.4)	1 652 (0.4)	0.01	1 189 (0.4)	1 212 (0.4)	0.001
Asian	12 263 (3.3)	16 405 (4.4)	0.06	11 207 (3.7)	11 077 (3.7)	0.002
Black or African American	60 421 (16.4)	55 874 (15.0)	0.04	46 727 (15.5)	45 054 (14.9)	0.02
Native Hawaiian or Other Pacific Islander	1 938 (0.5)	1 303 (0.4)	0.03	1255 (0.4)	1 201 (0.4)	0.003
White	202 490 (54.8)	201 823 (54.0)	0.02	165 496 (54.8)	166 220 (55.0)	0.005
Other Race[f]	27 334 (7.4)	31 744 (8.5)	0.04	24 415 (8.1)	25 265 (8.4)	0.01
Cholesterol measured	55 339 (15.0)	63 470 (17.0)	0.02	45 582 (15.1)	49 781 (16.5)	0.02
Cholesterol, ≥ 200 mg/dL	5 978 (1.6)	6 063 (1.6)	3E-04	4 904 (1.6)	4 731 (1.6)	0.005
BMI documented	64 613 (17.5)	87 238 (23.3)	0.15	59 413 (19.7)	60 198 (19.9)	0.007
BMI, pediatric, ≥95th percentile for age	26 993 (7.3)	28 924 (7.7)	0.02	23 301 (7.7)	23 167 (7.7)	0.002
BMI, pediatric, 85th < 95th percentile for age	15 494 (4.2)	21 530 (5.8)	0.07	14 304 (4.7)	14 672 (4.9)	0.006
Primary hypertension	5 085 (1.4)	3 292 (0.9)	0.05	3 199 (1.1)	3 025 (1.0)	0.006
Family history of diabetes mellitus	4 655 (1.3)	3 433 (0.9)	0.03	3 191 (1.1)	3 077 (1.0)	0.004
Pure hypercholesterolemia	3 179 (0.9)	3 662 (1.0)	0.01	2 730 (0.9)	2 618 (0.9)	0.004
Long term use of systemic steroids	1 318 (0.4)	601 (0.2)	0.04	658 (0.2)	581 (0.2)	0.006
Other low birth weight newborn	1 523 (0.4)	1 702 (0.5)	0.007	1 294 (0.4)	1 265 (0.4)	0.001
Psoriasis	1 157 (0.3)	1 609 (0.4)	0.02	1 063 (0.4)	963 (0.3)	0.006
Extremely low birth weight newborn	1 019 (0.3)	1 244 (0.3)	0.01	902 (0.3)	876 (0.3)	0.002
Polycystic ovarian syndrome	1 075 (0.3)	1 017 (0.3)	0.004	867 (0.3)	802 (0.3)	0.004
Autoimmune thyroiditis	1 074 (0.3)	948 (0.3)	0.007	825 (0.3)	774 (0.3)	0.003
Celiac disease	932 (0.3)	882 (0.2)	0.003	752 (0.3)	678 (0.2)	0.005
Crohn's disease	889 (0.2)	678 (0.2)	0.01	626 (0.2)	581 (0.2)	0.003

© 2024 Miller MG et al. *JAMA Network Open*.

Chronic rheumatic heart diseases	636 (0.2)	322 (0.09)	0.02	335 (0.1)	313 (0.1)	0.002
Secondary hypertension	603 (0.2)	260 (0.07)	0.03	292 (0.1)	252 (0.08)	0.004
Ulcerative colitis	536 (0.2)	415 (0.1)	0.01	372 (0.1)	364 (0.1)	7E-04
Hyperthyroidism	465 (0.1)	299 (0.08)	0.01	286 (0.1)	280 (0.09)	6E-04
Systemic lupus erythematosus	297 (0.08)	155 (0.4)	0.02	169 (0.06)	139 (0.06)	0.007
Immune thrombocytopenic purpura	317 (0.09)	216 (0.6)	0.01	208 (0.07)	184 (0.6)	0.003
Rheumatoid arthritis	34 (0.04)	70 (0.02)	0.01	69 (0.02)	63 (0.02)	0.001
Autoimmune hepatitis	97 (0.03)	32 (0.01)	0.01	77 (0.03)	59 (0.02)	0.004
Multiple sclerosis	81 (0.02)	54 (0.01)	0.006	51 (0.02)	43 (0.01)	0.002
Sjögren syndrome	72 (0.02)	74 (0.02)	2E-04	40 (0.01)	31 (0.01)	0.003
Myasthenia gravis	48 (0.01)	27 (0.01)	0.006	24 (0.01)	26 (0.01)	7E-04
Ankylosing spondylitis	44 (0.01)	37 (0.01)	0.002	30 (0.01)	34 (0.01)	0.001
Sarcoidosis	31 (0.01)	17 (0.01)	0.005	15 (0.01)	14 (0.01)	5E-04
Systemic sclerosis	37 (0.01)	40 (0.1)	7E-04	30 (0.01)	28 (0.01)	7E-04
Infant of mother with gestational diabetes	26 (0.01)	26 (0.01)	1E-04	23 (0.01)	18 (0.01)	0.002

Abbreviaton: ORI, other respiratory infections; SMD, standardized mean difference; BMI, body mass index

[a] Patient characteristics up to 1 day prior to index infection

[b] Data are presented as number (percentage) of patients unless otherwise indicated.

[c] An SMD greater than 0.1 is a threshold recommended for declaring imbalance.
[d] Index event was diagnosis of COVID-19 or a nonviral encounter (an ophthalmic issue, acne, or fracture)
[e] Race and ethnicity were self-reported or provider observed and were included because they are reported to be associated with COVID-19 diagnosis and severity.
[f] Includes a patient if they identify as multi-racial (more than one race specified) or if the patient belongs to a race that is not listed in the aforementioned options.

SI conversion factors: To convert cholesterol to mmol/L, multiply values by 0.0259.

© 2024 Miller MG et al. *JAMA Network Open*.

eTable 7. Comparison of Risk of New Diagnoses of T2D in Patients Aged 10 to 19 Years at 1, 3, and 6 Months From the Same Day COVID-19 Diagnosis or Nonviral Visit

**eTable 7.** Comparison of Risk of New Diagnoses of T2DM in Patients ages 10-19 years at 1 month, 3 months, and 6 months from the Same Day of COVID-19 Diagnosis or Nonviral Visit[a]			
	**Patients with New Type 2 Diabetes,[b] No (%)**		
**Time since index infection, mo; number in matched cohorts**	**COVID-19 (n = 302 119)**	**Nonviral Visit (n = 302 119)**	**RR (95% CI)**
1	173 (0.06)	105 (0.04)	1.65 (1.30-2.10)
3	216 (0.07)	130 (0.04)	1.66 (1.34-2.07)
6	289 (0.10)	169 (0.06)	1.71 (1.42-2.07)

Abbreviations: ORI, other respiratory infection; RR, relative risk

[a] Includes encounters related to ophthalmic issues, acne, or fractures
[b] Patients with new documentation of T2DM by ICD-10 code

© 2024 Miller MG et al. *JAMA Network Open*.
