# Supplementary material for: SARS-CoV-2 Infection and New-Onset Type 2 Diabetes Among Pediatric Patients, 2020 to 2022
Source: JAMA Netw Open. 2024 Oct 14;7(10):e2439444. doi: 10.1001/jamanetworkopen.2024.39444 (PMC11581647; doi:10.1001/jamanetworkopen.2024.39444)
Supplement: Supplement 2. — Data Sharing Statement [file jamanetwopen-e2439444-s002.pdf]

Data Sharing Statement

Miller. SARS-CoV-2 Infection and New-Onset Type 2 Diabetes Among Pediatric Patients, 2020 to 2022. *JAMA Netw Open*. Published October 14, 2024.
doi:10.1001/jamanetworkopen.2024.39444

Data
Data available: No

Additional Information
Explanation for why data not available: No data are available. We used a cloud-based database and cannot download the data set. In addition, the database is constantly being upgraded with new information, so the actual data from which the analysis was done will not be available at a subsequent time. That is why we indicate when the database was accessed and which specific data set was used. The EMR data are deidentified and so individual data cannot be made available to us or anyone else.
